# The physiological role of macrophages in reproductive organs

**DOI:** 10.1002/rmb2.12637

**Published:** 2025-02-14

**Authors:** Osamu Yoshino, Yosuke Ono

**Affiliations:** ^1^ Department of Obstetrics and Gynecology University of Yamanashi Yamanashi Japan

**Keywords:** macrophages, ovaries, reproductive physiology, uterus

## Abstract

**Background:**

Macrophages are essential immune cells critical to reproductive physiology. They regulate key processes such as follicular development, ovulation, and luteinization in the ovaries. Macrophages are also involved in endometrial remodeling, immune tolerance, and placentation in the uterus.

**Methods:**

This review examined the biological characteristics of macrophages and their role in ovarian, uterine, and fallopian tube physiology. It focused on findings from both animal and human studies to provide a comprehensive understanding of macrophage functions.

**Main Findings:**

In the ovaries, M1 macrophages play a role in folliculogenesis and ovulation through the inflammatory and angiogenic pathways. Macrophages also maintain the corpus luteum and vascular integrity. In the uterus, macrophages regulate tissue repair and remodeling during the menstrual cycle and play a critical role in implantation by maintaining immune tolerance and supporting decidualization. Dysregulation of the M1/M2 balance can cause implantation failure. In the fallopian tubes, macrophages mediate tissue repair and immune responses. Macrophage polarization dynamically adapts to physiological and pathological conditions in all reproductive organs highlighting the functional plasticity of these cells.

**Conclusion:**

Macrophage polarization and functions are pivotal in maintaining reproductive health. Hence, understanding the role of macrophages in various reproductive organs provides a foundation for developing new therapies.

## 1 INTRODUCTION

Macrophages are critical cells of the immune system. These cells also significantly play roles in reproductive physiology. This review aimed to detail the biological characteristics of macrophages, primarily in the ovaries and uterus. Understanding the diverse functions of macrophages in various stages of the reproductive process is critical to the development of therapeutic strategies. While this review primarily focused on animal studies (mouse models), studies conducted on human participants have been referenced when available. As limitations due to ethical considerations often restrict extensive research in human samples, most of our understanding of macrophages in reproductive organs comes from animal models.

### 1.1 Basic role of macrophages

Macrophages originate from bone marrow‐derived precursors and are involved in the phagocytosis of foreign substances, antigen presentation, and regulation of inflammation. These cells are found in the ovaries, uterus, fallopian tubes, and mammary glands.^1^ Macrophages are also involved in the regulation of the hypothalamic–pituitary–gonadal axis. Macrophages polarize into different functional states in response to environmental signals.^2^, ^3^

Macrophages exhibit functional plasticity, polarizing into classically activated (M1) macrophages and alternatively activated (M2) macrophages. M2 macrophages are further categorized into subtypes (M2a, M2b, M2c, and M2d), each characterized by distinct surface markers, the type of cytokines secreted, and the functions.^4^

### 1.2 The role of M1 and M2 macrophages

Macrophage polarization dynamically shifts according to environmental stimuli, and has an essential role in regulating immune responses and maintaining tissue homeostasis.^5^ During the initial stages of infection, M1 macrophages are dominant for eliminating pathogens and promoting inflammation. By contrast, M2 macrophages are dominant for tissue repair phases, for suppressing inflammation, and promoting tissue regeneration.^3^, ^6^ Chronic inflammation may occur when M1 macrophages are overly activated, leading to tissue damage. In contrast, M2 macrophages promote tumor growth and metastasis in a tumor microenvironment.^7^, ^8^ M1 macrophages are activated by lipopolysaccharides (LPS) and interferon‐gamma (IFN‐γ) and secrete proinflammatory cytokines necessary for controlling infections. On the other hand, M2 macrophages are activated by IL‐4, IL‐13, and IL‐10 and regulate inflammation and promote tissue repair. M2 macrophages can be further subdivided into M2a, M2b, M2c, and M2d, each induced by different stimuli and having specific functions.^9^, ^10^ *M2a* macrophages are induced by IL‐4 and IL‐13, and promote tissue repair and angiogenesis. These cells produce anti‐inflammatory cytokines such as IL‐10. *M2b* macrophages are induced by immune complexes and toll‐like receptor ligands, secrete increased levels of IL‐10, creating an immunosuppressive environment. *M2c* macrophages are induced by IL‐10 and TGF‐βcells, and are essential for apoptotic cell clearance and maintaining an anti‐inflammatory environment. *M2d* macrophages are induced by IL‐6 and LIF, and promote angiogenesis and immune evasion.^9^, ^10^ However, very few studies have evaluated M2 macrophages subsets in female reproductive organs physiology.

## 2 THE ROLE OF MACROPHAGES IN OVARIAN PHYSIOLOGY

The ovaries support female reproductive functions such as follicular development, ovulation, and luteinization. Macrophages are essential throughout these processes, interacting with endothelial cells to regulate folliculogenesis, luteinization, and follicular atresia.

### 2.1 Macrophages in follicular development and atresia

Macrophages are the most abundant immune cells in the ovary. Macrophages are localized in the follicles, corpora lutea, and stromal tissue.^11^, ^12^, ^13^ During follicular development, the distribution and number of ovarian macrophages increases.^14^ Several macrophage‐derived factors, such as hepatocyte growth factor (HGF), epidermal growth factor (EGF), and basic fibroblast growth factor (FGF), can affect follicular development.^15^, ^16^

#### 2.1.1 Animal studies

In mouse models, various macrophage depletion experiments were conducted to understand the role of these cells. Van der Hoek et al. reported that partial depletion of ovarian macrophages using clodronate liposomes inhibited folliculogenesis and significantly reduced ovulation rates.^14^ Similarly, the use of CD11b‐diphtheria toxin receptor transgenic (DTR) mice allowed selective depletion of CD11b‐positive cells (a pan‐macrophage marker), highlighting the role of macrophages in ovarian functions. Diphtheria toxin (DT) injection can rapidly deplete pan‐macrophages.^17^ Turner et al. demonstrated that pan‐macrophage depletion during folliculogenesis resulted in ovarian hemorrhage with reduced endothelial cells and follicular atresia.^17^

##### Folliculogenesis

**FIGURE 1:**
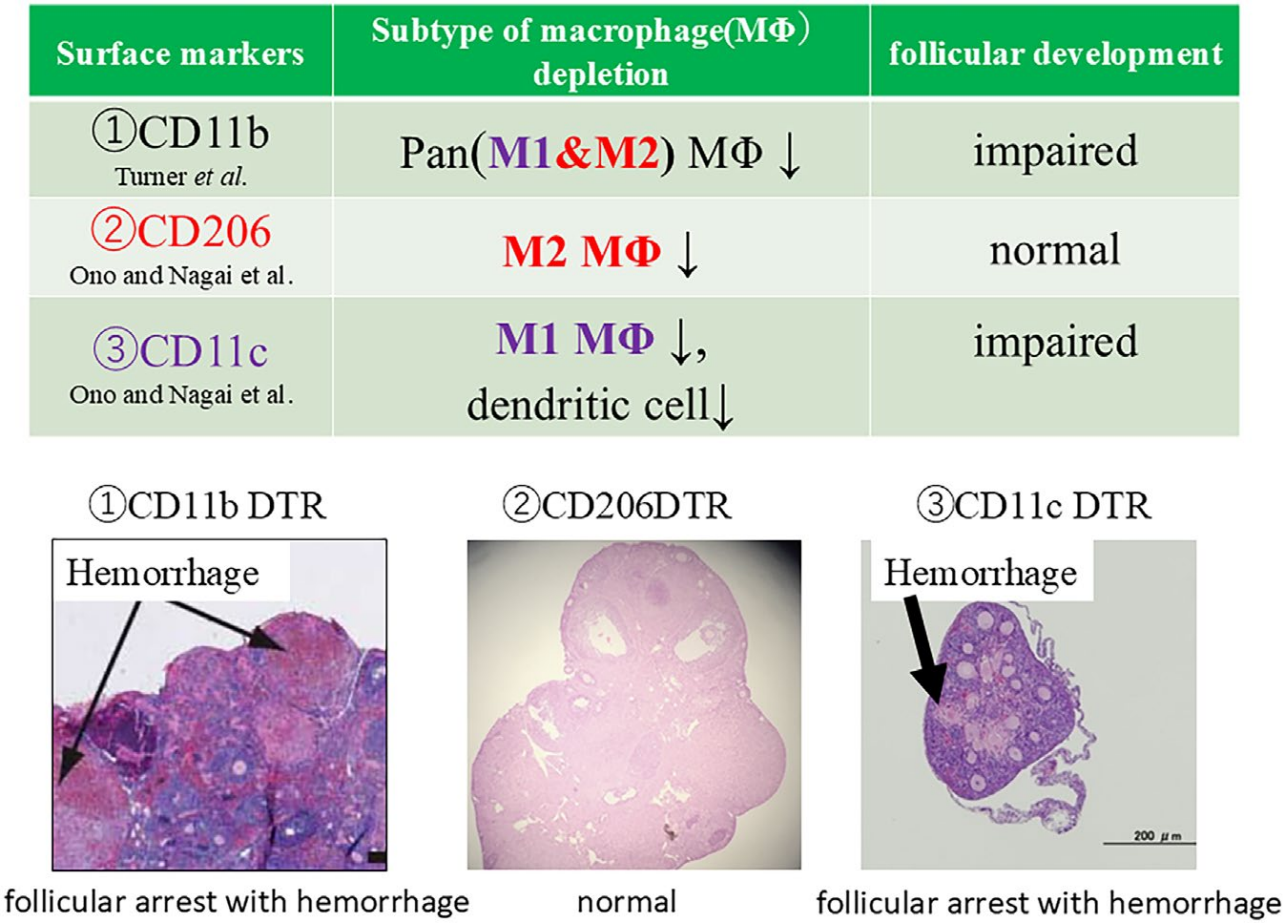
Phenotypes of follicular development observed in various DTR mice. Phenotypes of ①CD11b DTR mice, pan (M1 + M2) macrophage depletion model ②CD206 DTR mice, M2 macrophage depletion model ③CD11c DTR mice, M1 macrophage and dendric cell depletion model. In CD11b and CD11c DTR mice, follicular arrest with hemorrhage was observed.

**FIGURE 2:**
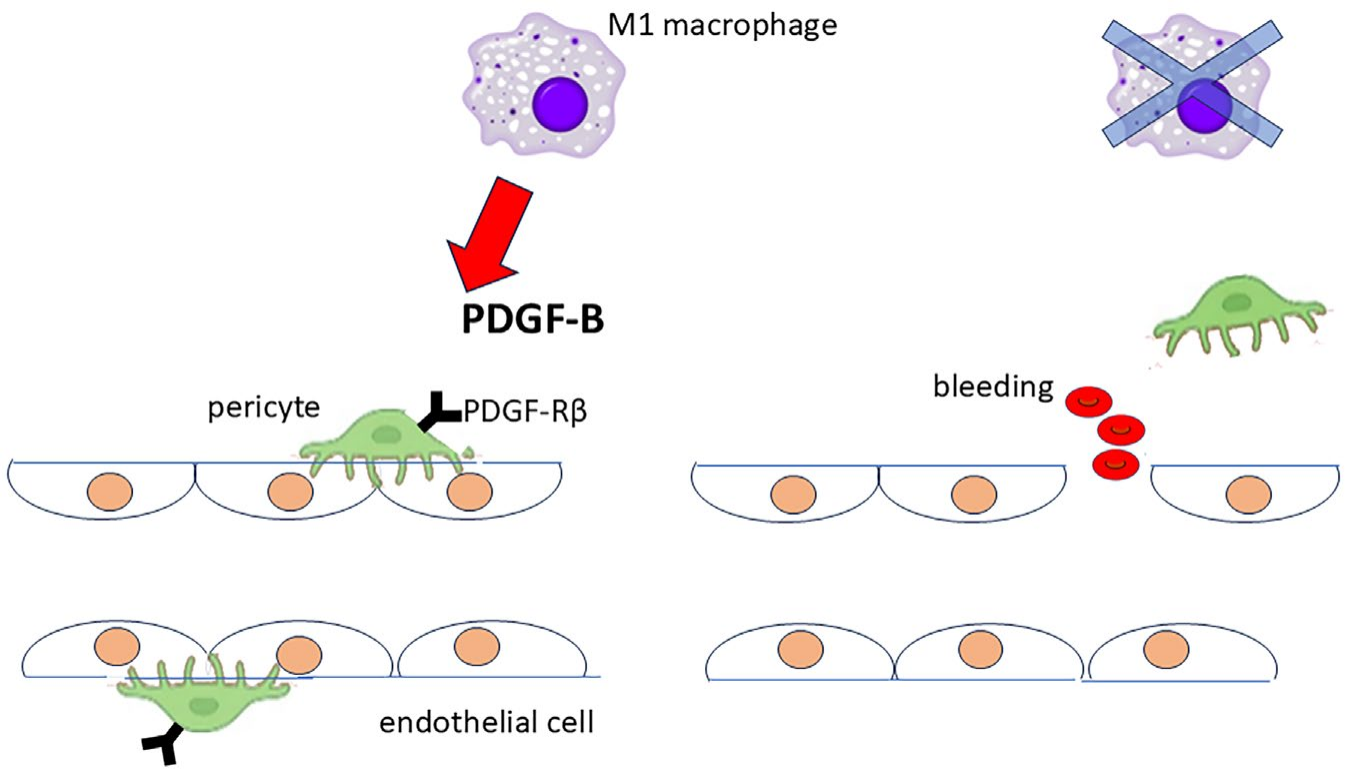
Interaction between pericytes and endothelial cells for maintaining vascular integrity. M1 macrophages produce the angiogenic factor PDGF‐B which mobilizes pericytes through PDGF‐Rβ signaling. Both pericytes and endothelial cells are embedded within the basement membrane of microvessels. In the absence of M1 macrophages, the interaction between pericytes and endothelial cells is disrupted leading to vascular instability and hemorrhage.

Macrophages can be broadly classified as M1 (proinflammatory) or M2 (alternatively activated).^10^ In mouse studies, M1 macrophages are known to activate primordial follicles, whereas M2 macrophages maintain follicles in a dormant state.^18^ Our research, using a gonadotropin‐primed mouse model showed an increase in CD11c‐positive M1 macrophages in ovaries whereas CD206‐positive M2 macrophages did not increase.^19^ Furthermore, depletion of CD206‐positive M2 macrophages using CD206 DTR mice did not impair follicle formation, and ovulation, fertilization, and implantation rates were similar to that observed in wild‐type mice.^19^ In contrast, CD11c DTR mice, which can deplete M1 macrophages and dendritic cells, exhibited ovarian hemorrhage with impaired folliculogenesis (Figure 1) and reduced staining for the pericyte marker, platelet‐derived growth factor (PDGF)‐receptor β^20^ and the endothelial cell marker (CD34).^19^ M1 macrophages are known to produce PDGF‐B, which induces pericytes and enhances vascular function through interactions with endothelial cells (Figure 2).^21^ Our study suggested that depletion of CD11c‐positive cells led to decreased endothelial–pericyte interactions causing hemorrhage. Pan‐macrophage depletion can cause ovarian hemorrhage and halted folliculogenesis.^17^ Furthermore, depletion of CD11c‐positive cells (M1 macrophages and dendritic cells) resulted in a similar phenotype, indicating that M1 macrophages (and not M2 macrophages) are involved in folliculogenesis.^19^ Other studies have also showed that conditional knockout of CD11c within the mouse ovary induces extensive cell death in growing follicles.^22^ It is worth noting that, in addition to M1 macrophages, dendritic cells (DCs) also express CD11c. Therefore, it is possible that DCs are involved in follicular development in CD11c‐DTR mice. Further investigation is required to understand the role of DCs.

##### Atretic follicles

The infiltration and recruitment of macrophages into atretic follicles are primarily mediated by IL‐33, which is produced by endothelial cells adjacent to atretic follicle.^23^, ^24^ After macrophage invasion, macrophages participate in the degradation of atretic follicles by secreting matrix metalloproteinase‐3 (MMP‐3).^25^ Interestingly, a deficiency in the Il33 gene disrupts the normal clearance of atretic follicles, leading to the accumulation of tissue debris rich in age‐related metabolic waste such as lipofuscin. This accelerates ovarian aging and functional decline, reducing the reproductive lifespan of control mice by two‐thirds. These data suggest that proper regulation of tissue and cellular waste removal plays a crucial role in maintaining tissue integrity and preventing aging.^23^

#### 2.1.2 Human studies

Human studies investigating macrophages in normal human ovarian physiology are limited, but many studies have been conducted using PCOS (polycystic ovary syndrome) patients. It is known that there is a strong shift toward M1 macrophages in the pathology of PCOS. In the peripheral blood and ovaries of patients with PCOS, the number of M1 macrophages increases,^26^ and the M1: M2 ratio is higher than that in controls.^27^ It has been reported that women with PCOS have an increased number of preantral and antral follicles. In mice models, M1 macrophages activate primordial follicles^18^ and it is possible that the excessive number of M1 macrophages in human PCOS might caused increased follicular count. Further research in human participants is required for a better understanding of macrophages in reproductive physiology.

### 2.2 Role of macrophages in ovulation

Ovulation is an inflammatory response that is known to be suppressed by nonsteroidal anti‐inflammatory drugs (NSAIDs). Preovulatory follicles exhibit increased levels of cytokines and chemokines, such as TNF‐α, IL‐1β, IL‐6, IL‐8, and granulocyte‐colony stimulating factor (G‐CSF).^28^, ^29^, ^30^ These factors induce the accumulation of neutrophils and macrophages around granulosa cells, producing proteases that disrupt the follicular wall and promote ovulation.

#### 2.2.1 Animal studies

Preovulatory macrophages secrete proteases and enzymes to promote the breakdown of the follicular wall and recruit neutrophils. After ovulation, macrophages remove follicular debris and secrete tissue repair factors to support luteinization.^1^ Indeed, it has been reported that the depletion of neutrophils using antibodies reduces ovulation rates.^31^ Mouse experiments using clodronate liposomes have showed that depletion of macrophages can inhibit rupture of the follicular wall.^14^

Colony‐stimulating factor 1 (CSF‐1) is a hematopoietic growth factor necessary for the mobilization, proliferation, and differentiation of mononuclear phagocytes.^32^ CSF‐1‐deficient osteopetrotic (*Csf1* ^*op*^ */Csf1* ^*op*^) mice exhibit reduced macrophages.^33^ Compared to wild‐type female mice, *Csf1* ^*op*^ */Csf1* ^*op*^ mice have abnormal ovarian phenotypes such as disrupted ovarian cycles, reduced ovulation rates, and impaired steroidogenesis associated with decreased LH levels.^34^ Although ovulation rates in these mice are significantly lower than that in wild‐type mice, the implantation rate of fertilized eggs is normal. These effects are thought to result from the central nervous system rather than local, ovarian abnormalities due to CSF‐1 deficiency.^33^, ^34^

In mice experiments using clodronate liposomes, depletion of pan‐macrophages suppressed rupture of the follicular wall.^14^ However, it is not clear which subtype of macrophages, M1 or M2 is responsible for ovulation. Cohen et al. demonstrated that depletion of M1 macrophages and dendritic cells using CD11c DTR mice inhibited ovulation.^26^ Specifically, that study focused on dendritic cells and showed that depletion of CD11c‐positive cells suppressed cumulus expansion of granulosa cells and inhibited ovulation. This effect was restored by adding dendritic cells.^35^ However, the role of M1 macrophages in the process of ovulation was not evaluated. As discussed above, a depletion of CD206‐positive M2 macrophages using CD206 DTR mice did not impair follicle formation, and ovulation, fertilization, and implantation rates remained comparable to that of the wild‐type mice.^19^ Since the ovulation process involves inflammatory reactions, its is possible that M1 macrophages contribute to ovulation. However, this needs to be examined in further studies.

#### 2.2.2 Human studies

Studies using human samples have shown an upregulation of MCP‐1 in the stromal compartment around the follicles during the ovulatory process and a high density of macrophages were found in the earlier phases of ovulation indicating that inflammation‐like reactions are essential to the ovulatory process.^36^ A recent study investigated the single‐cell transcriptome of the follicular microenvironment around metaphase‐II oocytes in human preovulatory follicles and found that follicular macrophages are involved in immune responses, extracellular matrix remodeling, and assist granulosa cells in promoting the resumption of oocyte meiosis.^37^

### 2.3 The role of macrophages in luteinization

Corpus luteum is temporarily formed after ovulation and secretes progesterone, which is essential for endometrial proliferation and maintenance of pregnancy. Macrophages are crucial for luteinization and for corpus luteum functions. During luteinization, macrophages secrete vascular endothelial growth factor (VEGF) and other growth factors, which form a vascular network within the corpus luteum.^38^ This vascular network supplies the necessary nutrients and oxygen to promote progesterone production.^38^ Additionally, macrophages maintain avascular network in the corpus luteum ensuring a normal function and progesterone secretion. Dysfunction of the corpus luteum can lead to early miscarriage and infertility, and macrophage dysfunction may impair luteinization and its maintenance potentially causing progesterone deficiency.^39^

#### 2.3.1 Animal studies

Care et al. used a pan macrophage depletion model to show that macrophages, via the production of the endothelial growth factors VEGF‐C and VEGF‐D, maintained an extensive vascular network within the corpus luteum supporting the production of progesterone necessary for pregnancy maintenance.^39^

A study using bovine models showed that shortly after ovulation, pericytes might aid the outgrowth of endothelial cells in the bovine corpus luteum.^40^

Luteal regression is triggered by prostaglandin F2α (PGF2α).^39^ Ovarian macrophages polarize to the M1 phenotype, and secrete TNF‐α, which promotes PGF2α production and luteolysis. This indicates that the M1 macrophage subset plays an important role in luteal regression.^41^

#### 2.3.2 Human studies

Luteal dysfunction can contribute to early miscarriage and infertility, and macrophage dysfunction may impair the formation and maintenance of the corpus luteum potentially leading to progesterone deficiency. Macrophages and endothelial cells are closely associated with luteal cells. Macrophages produce IL‐1β, TNF‐α, and reactive oxygen species, which decrease progesterone production by luteal cells.^42^, ^43^, ^44^ The function of endothelial cells in the corpus luteum may also be affected potentially leading to decreased expression of molecules such as VEGF, which promote endothelial cell survival.^45^

#### 2.3.3 The role of macrophages in vascular integrity

Overall, macrophages and endothelial cells are deeply involved in follicular development, corpus luteum formation, and follicular atresia. Additionally, pericytes are involved in stabilizing the vascular network for follicular development and the formation and maintenance of the corpus luteum.^21^ Macrophages produce the angiogenic factor PDGF‐B, which mobilizes pericytes via PDGF‐Rβ.^46^ Pericytes possess a cell body with a prominent nucleus and small content of cytoplasm, with several long processes embracing the endothelium wall.^47^ Pericytes and endothelial cells are embedded within the basement membrane of microvessels.^48^ Adhesion between pericytes and endothelial cells is promoted by TGF‐β secreted from both cell types in a paracrine and autocrine manner.^49^ Pericytes communicate with endothelial cells by direct physical contact and paracrine signaling pathways.^50^ The pericytes interact with endothelial cells to establish vascular integrity.^21^ We hypothesized that disruption of this interaction leads to hemorrhage (Figure 2).^19^ In fact, Kuhnert et al. reported that inhibiting PDGF‐Rβ with a decoy receptor induced hemorrhage within the ovary.^51^ Similarly, PDGF‐Rβ inhibitor caused severe bleeding in the ovary due to selective pericyte loss within the corpus luteum leading to luteal regression.^52^ It has also been shown that PDGF‐B mutant embryos suffer fatal hemorrhages just before birth.^53^ Collectively, we estimated that macrophages contribute to the ovarian function by producing PDGF‐B to induce the stability of pericytes and endothelial cells to maintain blood flow, and contribute to follicular development and the maintenance of the corpus luteum.^19^

### 2.4 Ovarian aging

Currently, in infertility treatment, the aging of oocytes and ovaries has become a significant issue. While oocyte and ovarian freezing is currently one method to halt aging, future treatments based on the mechanisms of aging are expected to emerge. It appears that macrophages also play a role in ovarian aging. Although ovarian aging encompasses changes in multiple cellular and structural components of the ovary including oocytes, follicular somatic cells, stromal cells, blood vessels, and extracellular matrix, this section focuses specifically on the role of macrophages in stromal aging and their interactions within the stromal microenvironment.

#### 2.4.1 Animal studies

It has been shown that macrophages play an important role in ovarian aging. It seems that there is still no consensus on whether M1 or M2 macrophages are more involved in ovarian aging. Xiao et al. reported that in 10‐month‐old mice, the proportion of M1 macrophages in the ovaries increases compared to younger mice, and the expression of proinflammatory genes, such as IL‐6, TNF‐α, and iNOS, is elevated.^18^ Umehara T et al. showed that M2 macrophages are more prevalent in the ovarian stroma of aged mice, and fibrosis is caused by mitochondrial dysfunction. Furthermore, antifibrotic drugs decrease M2 macrophage polarization and restore ovulation in aged mice.^54^

#### 2.4.2 Human studies

It has been shown that the number of macrophages in the ovarian stroma increases significantly in postmenopausal women. These macrophages produce high levels of IL‐16, and exposure to FSH significantly increases the expression of nuclear IL‐16. This suggests that ovarian macrophages play an inflammatory role in the aging process of women.^55^ Further human studies are needed to elaborate on the role of different macrophage phenotypes in ovarian aging.

## 3 THE ROLE OF MACROPHAGES IN UTERINE PHYSIOLOGY

The uterus provides a complex environment that supports embryo implantation and development. Macrophages play crucial roles in endometrial remodeling during the menstrual cycle and early pregnancy, maintaining immune tolerance, and supporting placental formation.^56^

### 3.1 Menstrual cycle and macrophages

The endometrium undergoes dynamic changes in response to the menstrual cycle, repeating phases of proliferation, secretion, and menstruation. During these processes, macrophages play key roles in tissue repair, regeneration, and immune response regulation. M1 macrophages mainly handle inflammation and tissue clearance, whereas M2 macrophages support tissue repair and regeneration. Factors such as estrogen, progesterone, and granulocyte–macrophage colony‐stimulating factor (GM‐CSF) regulate these processes, ensuring appropriate immune responses and tissue remodeling.^9^, ^57^

#### 3.1.1 Proliferative phase

The endometrium thickens under the influence of estrogen. Macrophages promote angiogenesis and assist endometrial regeneration primarily showing the M2 phenotype and secreting anti‐inflammatory cytokines to support tissue repair and regeneration.^9^, ^58^, ^59^, ^60^

#### 3.1.2 Secretory phase

Progesterone influences the endometrium to mature and prepare for pregnancy. Macrophages become activated, supporting immune regulation and tissue maturation. During this phase, M2 macrophages maintain an anti‐inflammatory environment, stabilizing the tissue.^9^, ^58^

#### 3.1.3 Menstrual phase

The endometrium sheds during menstruation. Macrophages increase in number and lead to tissue clearance and repair. During this phase, M1 macrophages secrete proinflammatory cytokines, managing tissue clearance and inflammation control.^9^ During menstruation, the endometrium resembles a “wound” and the wound‐healing process is crucial for maintaining reproductive function.^60^ The number of uterine macrophages peaks during menstruation as the progesterone levels decrease.^61^ The apoptotic endometrial cells that are shed during menstruation are phagocytosed by macrophages.^62^ Additionally, macrophages express matrix metalloproteinases, which are upregulated during menstruation and contribute to tissue breakdown during the menstrual process.^63^

### 3.2 Effects of hormones on macrophages

Estrogen significantly influences the dynamics and functions of macrophages. Estrogen regulates the phenotype of macrophages at each stage of the menstrual cycle, enabling appropriate immune responses and tissue remodeling. GM‐CSF plays a crucial role in the proliferation and activation of macrophages, and its secretion is regulated throughout the menstrual cycle to optimize macrophage function.^59^ Estrogen action is mediated by intracellular estrogen receptors (ERs) (namely, ERα and ERβ) and by G protein‐coupled estrogen receptor 1 (a plasma membrane protein).^64^ A previous study has reported that endometrial macrophages express estrogen‐related receptor beta suggesting that these cells are regulated in an estrogen‐dependent manner.^65^ However, the expression of estrogen receptors in macrophages is a topic of debate. RNA sequencing data from three datasets (two mouse and one human), which was derived from peritoneal macrophages did not detect ERβ, whereas mRNA expression of ERα and G protein‐coupled estrogen receptor 1 were observed.^66^ Interestingly, it has been reported that estrogen promotes M2 phenotype during cutaneous repair, which is an alternatively activated type macrophage, through estrogen receptor α.^67^

Uterine macrophages do not express progesterone receptors indicating that they are indirectly regulated through factors secreted by progesterone‐responsive endometrial cells.^68^ According to the aforementioned databases, peritoneal macrophages derived from mice and humans do not exhibit the expression of progesterone receptors.^66^

### 3.3 Role of macrophages in early pregnancy

In the uterus, macrophages are recruited to the endometrium in response to seminal fluid and early pregnancy signals during the peri‐implantation period.^69^, ^70^ Uterine macrophages are involved in the remodeling process and induce the expression of epithelial glycoproteins necessary for embryo implantation.^71^ They also contribute to decidualization and trophoblast invasion of the placenta.^72^, ^73^

#### 3.3.1 Animal studies

Zhang and colleagues summarized the transition from M1 to M2 macrophages during pregnancy.^73^ During the implantation phase, activated M1 macrophages produced proinflammatory cytokines such as IL‐1β, TNF‐α, and nitric oxide (NO) which promote an inflammatory response necessary for embryo implantation. As the trophoblasts invade the uterine stroma, decidual macrophages exhibit both M1 and M2 profiles, a state that persists until the early second trimester. This dual profile helps prevent embryo rejection while promoting trophoblast invasion and vascular remodeling. To support fetal development, an M2‐dominant environment is established in the uterus by the end of pregnancy.^73^

**FIGURE 3:**
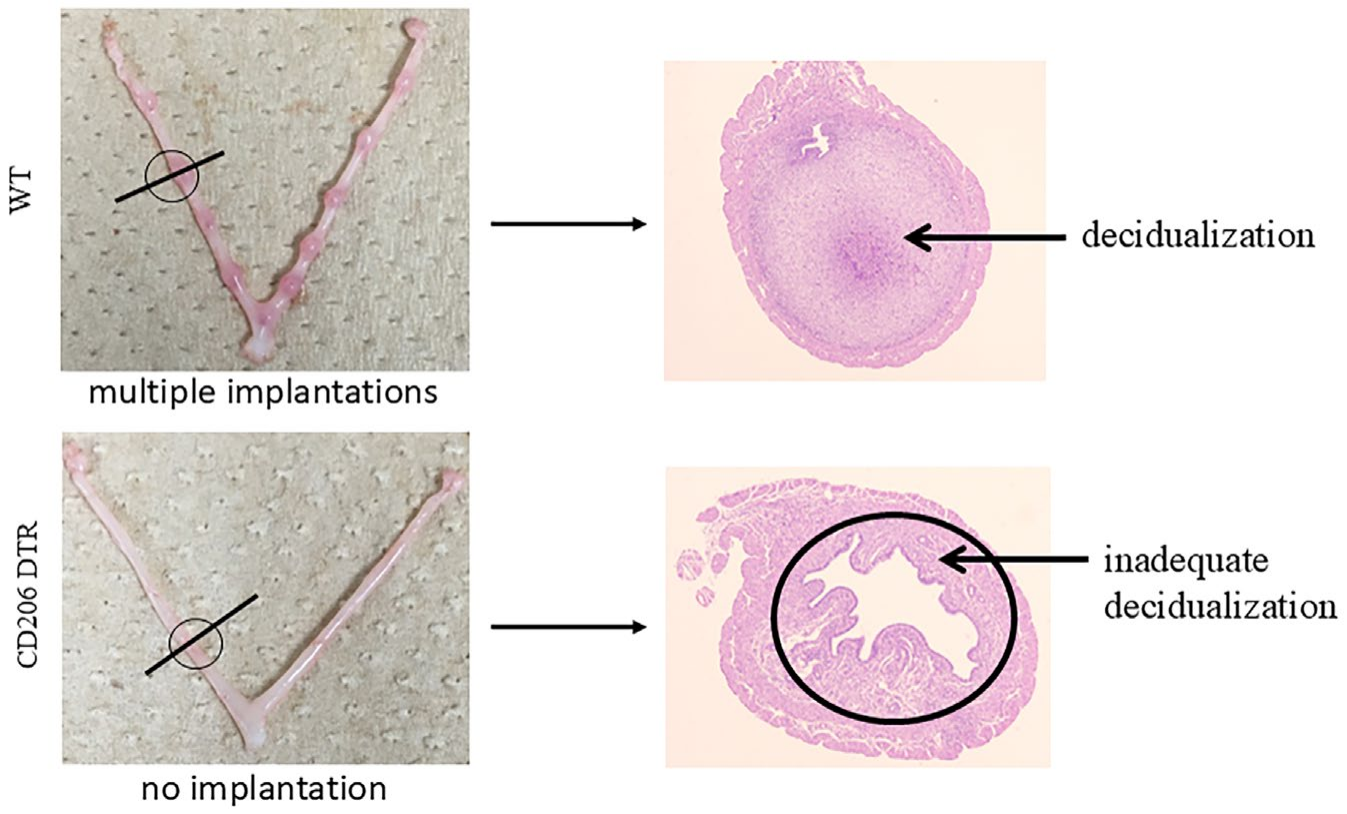
Impact of M2 macrophage depletion on uterine implantation and decidualization. In CD206 DTR mice lacking M2 macrophages, no implantation sites were observed in the uterus (lower panel). Compared to wild‐type mice (upper panel), CD206 DTR mice exhibited impaired decidualization of endometrial stromal cells.

**FIGURE 4:**
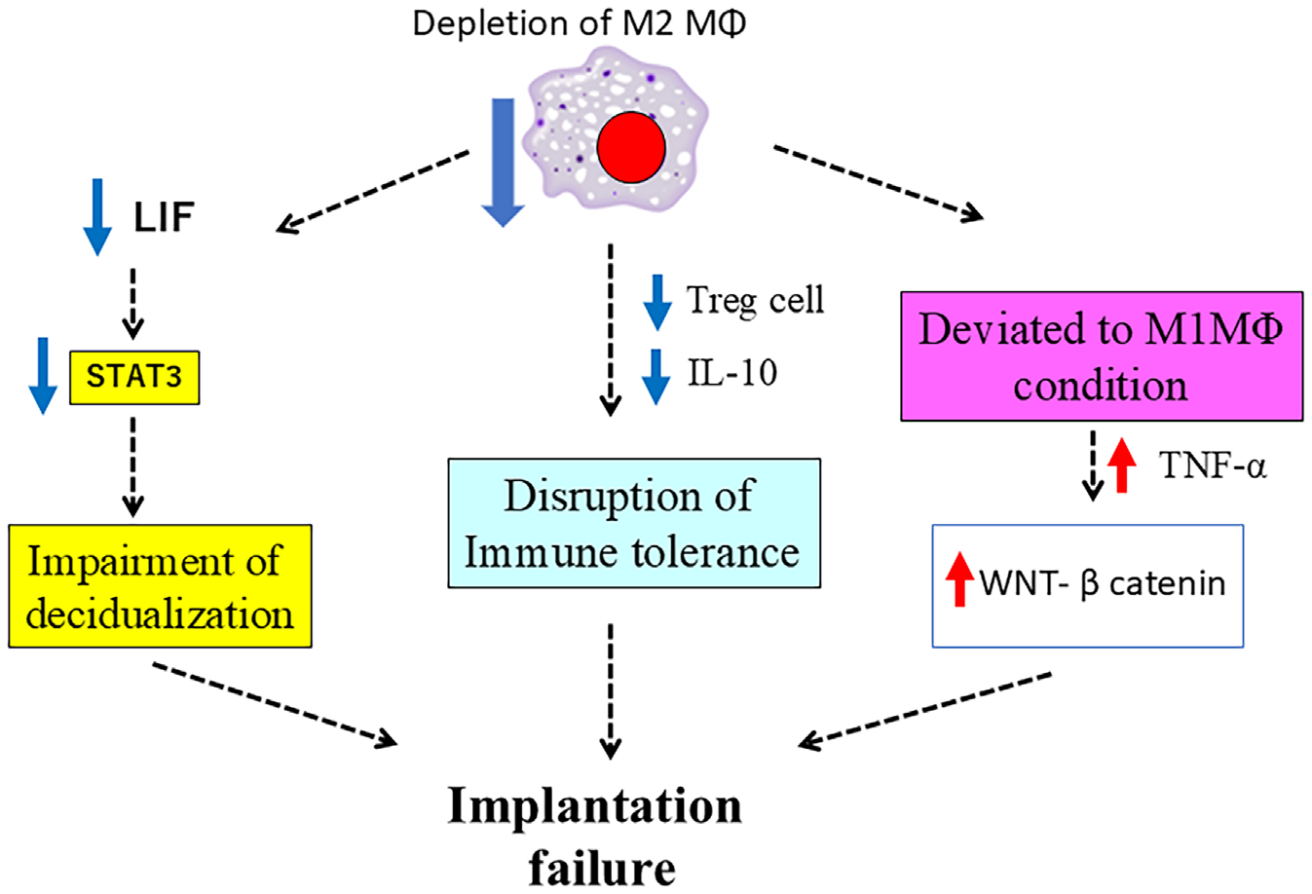
The mechanism of impaired implantation in M2 macrophage depleted mouse. Depletion of M2 macrophages suppressed the leukemia inhibitory factor (LIF)‐JAK‐STAT3 pathway. M2 macrophages induced regulatory T cells (Treg),^78^ and Treg depletion reduced IL‐10 disrupting immune tolerance. The absence of M2 macrophages led to an excessive increase in M1 macrophages inducing abnormal TNF‐α expression and activating the WNT‐β‐catenin pathway in the uterine epithelium.

In experiments using CD206DTR mice, we proved that the depletion of M2 macrophages during the implantation phase resulted in implantation failure (Figure 3).^74^ The mechanisms of implantation failure are described in Figure 4. An absence of M2 macrophages led to an excessive increase in M1 macrophages inducing abnormal TNF‐α expression and activating the WNT‐β‐catenin pathway in the uterine epithelium. This caused sustained proliferation of epithelial cells and the impairment of decidualization resulted in implantation failure (Figure 3).^74^ Furthermore, the depletion of M2 macrophages suppressed the leukemia inhibitory factor (LIF)‐JAK‐STAT3 pathway which is crucial for implantation,^75^ thus preventing the endometrium from becoming receptive to the embryo.^74^ Other groups have also reported that M2 macrophages contribute to implantation through various mechanisms. The induction of immune tolerance, including regulatory T cells (Tregs) and IL‐10 is essential for implantation.^76^, ^77^ M2 macrophages induce Tregs^78^ and their depletion reduces IL‐10, disrupting immune tolerance and leading to implantation failure.^76^ LIF is an essential molecule involved in implantation, partly by chemotactic recruitment of macrophages. In LIF knockout mice, which exhibit implantation failure, macrophages in the uterus are reported to decrease by more than 50%, highlighting the close relationship between macrophages and LIF.^79^

#### 3.3.2 Human studies

Our study based on human samples also showed that the proportion of M1 macrophages and TNF‐α expression in the endometrium of patients with implantation failure was significantly higher suggesting that correcting the M1/M2 macrophage balance could be a potential indicator for treating implantation failure.^74^ Similarly, recent clinical studies have demonstrated that the proportion of CD68+ (M1 + M2) and CD163+ (M2) macrophages in the endometrium during the midsecretory phase is significantly associated with IVF‐ET outcomes.^80^

## 4 FALLOPIAN TUBES

Fallopian tubes are essentials component of the female reproductive system and connect the ovaries to the uterus. They play a pivotal role in fertilization and the subsequent implantation of the embryo by facilitating the movement of gametes.^81^ Fallopian tubes are known to be affected by various pathological conditions such as infections and endometriosis. Consequently, the development of tubal disease has a relationship with infertility. There are very few animal studies conducted using mice that have investigated the role of macrophages in the fallopian tubes. The below presented studies have primarily focused on humans.

### 4.1 Presence and distribution of macrophages

Macrophages are primarily located in the epithelium and lamina propria,^82^ where they contribute to tissue repair and immune responses. Flow cytometric analysis of immune cells from fimbria specimens showed that the predominant cell type was M1 macrophages, followed by Tregs, CD8 cytotoxic T cells and M2 macrophages.^83^ Others found the presence of macrophage in the human tubal epithelium to be minimal.^84^

### 4.2 Changes in macrophages during the menstrual cycle

Studies have reported variations in the number of macrophages during the menstrual cycle, with some reporting an increase in the number of macrophages during the secretory phase.^82^, ^85^ Ovulation is an inflammatory response, following which, the fallopian tubes are exposed to the follicular fluid which is enriched with inflammatory mediators.^28^, ^29^, ^30^ Therefore, these inflammatory mediators increase the number of macrophages in the tubal walls.^86^

### 4.3 Macrophages in fallopian tube pathologies

In conditions such as salpingitis and hydrosalpinx, an increase in the number of macrophages has been reported.^87^, ^88^ In particular, CD68‐positive M1 macrophages were predominant in salpingitis and were shown to produce proinflammatory cytokines (IL‐6 and IL‐8).^88^ Several studies have confirmed that the number of macrophages significantly increases in ectopic pregnancies when compared to normal fallopian tubes.^88^, ^89^ Proinflammatory cytokines (IL‐6 and IL‐8), derived from macrophages, activate the expression of implantation‐associated molecules and Wnt signaling pathway predisposing the tubal epithelium to an adhesive and receptive state for embryo implantation.^88^

## 5 FUTURE DIRECTIONS

Targeting specific macrophage subtypes has the potential for identifying new therapeutic strategies aimed at improving outcomes in folliculogenesis and implantation disorders. Nanotechnology‐based drug delivery systems are also gaining attention. This review demonstrated that research an understanding of ovarian physiology has advanced primarily by using clodronate in mouse experiments leading to a reduction of pan‐macrophages. This treatment can is classified under nanotechnology therapeutics. The modulation of macrophages by clodronate is effective not only for physiological studies but also in mouse models of obesity,^90^ melanoma,^91^ and endometriosis.^92^ However, further investigation is needed for human application. Targeted therapy that employs nanoparticles to specific macrophages could enhance treatment efficacy while minimizing side effects.^88^ This approach may enable effective therapy with minimal side effects. In this review, we divided macrophages into M1 or M2 macrophages, and assessed reproductive phenomena from the points of deviation to M1 or M2 macrophages. Modulating macrophage subtypes may offer new therapeutic strategies for folliculogenesis and implantation disorders. In cases of excessive M1 macrophages, treatment with G‐CSF to induce M2 macrophages has been evaluated.^93^ Conversely, in M2 macrophage‐dominant conditions, GM‐CSF treatment may shift macrophages to the M1 phenotype.^93^ In this review, we introduced the subclassification of M2 macrophages into M2a, M2b, M2c, and M2d. It may be necessary to evaluate M2 macrophages further based on this reclassification. Additionally, while we presented data by broadly classifying macrophages into M1 and M2 categories, it has been said that this M1/M2 classification fails to fully capture the characteristics of macrophages.^94^ In the future, macrophage‐targeted therapies might be useful in personalized medicine, and a more precise classification of macrophages is essential.

## 7 CONFLICT OF INTEREST STATEMENT

We have no conflicts of interest for this article.
